# Heart Muscle Microphysiological System for Cardiac Liability Prediction of Repurposed COVID-19 Therapeutics

**DOI:** 10.3389/fphar.2021.684252

**Published:** 2021-08-04

**Authors:** Bérénice Charrez, Verena Charwat, Brian A. Siemons, Ishan Goswami, Courtney Sakolish, Yu-Syuan Luo, Henrik Finsberg, Andrew G. Edwards, Evan W. Miller, Ivan Rusyn, Kevin E. Healy

**Affiliations:** ^1^ Department of Bioengineering and California Institute for Quantitative Biosciences (QB3), University of California at Berkeley, Berkeley, CA, United States; ^2^ Department of Veterinary Integrative Biosciences, College of Veterinary Medicine and Biomedical Sciences, Texas A&M University, College Station, TX, United States; ^3^ Simula Research Laboratory, Oslo, Norway; ^4^ Department of Pharmacology, School of Medicine, University of California at Davis, Davis, CA, United States; ^5^ Department of Chemistry, University of California at Berkeley, Berkeley, CA, United States; ^6^ Department of Molecular and Cell Biology, University of California at Berkeley, Berkeley, CA, United States; ^7^ Helen Wills Neuroscience Institute, University of California at Berkeley, Berkeley, CA, United States; ^8^ Department of Materials Science and Engineering, University of California at Berkeley, Berkeley, CA, United States

**Keywords:** microphysiological systems, induced pluripotent stem cells, heart muscle, cardiomyocytes, SARS-CoV-2, hydroxychloroquine, azithromycin, polytherapy

## Abstract

Despite global efforts, it took 7 months between the proclamation of global SARS-CoV-2 pandemic and the first FDA-approved treatment for COVID-19. During this timeframe, clinicians focused their efforts on repurposing drugs, such as hydroxychloroquine (HCQ) or azithromycin (AZM) to treat hospitalized COVID-19 patients. While clinical trials are time-consuming, the exponential increase in hospitalizations compelled the FDA to grant an emergency use authorization for HCQ and AZM as treatment for COVID-19, although there was limited evidence of their combined efficacy and safety. The authorization was revoked 4 months later, giving rise to controversial political and scientific debates illustrating important challenges such as premature authorization of potentially ineffective or unsafe therapeutics, while diverting resources from screening of effective drugs. Here we report on a preclinical drug screening platform, a cardiac microphysiological system (MPS), to rapidly identify clinically relevant cardiac liabilities associated with HCQ and AZM. The cardiac MPS is a microfabricated fluidic system in which cardiomyocytes derived from human induced pluripotent stem cells self-arrange into a uniaxially beating tissue. The drug response was measured using outputs that correlate with clinical measurements such as action potential duration (proxy for clinical QT interval) and drug-biomarker pairing. The cardiac MPS predicted clinical arrhythmias associated with QT prolongation and rhythm instabilities in tissues treated with HCQ. We found no change in QT interval upon acute exposure to AZM, while still observing a significant increase in arrhythmic events. These results suggest that this MPS can not only predict arrhythmias, but it can also identify arrhythmias even when QT prolongation is absent. When exposed to HCQ and AZM polytherapy, this MPS faithfully reflected clinical findings, in that the combination of drugs synergistically increased QT interval when compared to single drug exposure, while not worsening the overall frequency of arrhythmic events. The high content cardiac MPS can rapidly evaluate the cardiac safety of potential therapeutics, ultimately accelerating patients’ access to safe and effective treatments.

## Introduction

With the high incidence of COVID-19 cases worldwide, almost 120 million people have tested positive, leading to more than 3.7 million deaths by June 2021 (JHU, 2020). Global deployment of vaccines remains a challenge that may take as long as 12 months to address (Burgos et al., 2021; Randall, 2021). It is therefore critical to continue to treat COVID-19 patients with appropriate therapeutics, some of them repurposed from other clinical indications. Early clinical trials identified hydroxychloroquine (**HCQ**) and azithromycin (**AZM**) as promising therapeutics to help treat or reduce the effects of SARS-CoV-2 (Gautret et al., 2020; Huang et al., 2020; Wang et al., 2020; Yao et al., 2020). On March 28th, 2020 the FDA granted an emergency use authorization for oral administration of HCQ as treatment against COVID-19 (FDA, 2020a). In early June 2020 the FDA cautioned the use of HCQ for COVID-19 treatment outside of hospital settings due to the reported risk of heart complications (FDA, 2020b). The authorization for HCQ and AZM was revoked on June 22, 2020, largely due to the lack of evidence for their effectiveness in COVID-19 patients and the risk of side effects (Furtado et al., 2020; Gérard et al., 2020; Molina et al., 2020; Rosenberg et al., 2020; Self et al., 2020). This sequence of events was politically and scientifically controversial and illustrated important challenges that can arise with emergency drug authorizations during crises, notably the authorization of potentially ineffective or unsafe therapeutics, while diverting resources from screening of effective drugs (Thomson and Nachlis, 2020). Close to 250 clinical trials were started to study the efficacy of HCQ treatment for COVID-19, 80 of them a polytherapy by combining HCQ with the antibiotic AZM. Only 11 polytherapy trials were completed, while several trials were terminated due to adverse cardiac side effects associated with the treatments (Andrea Natale, 2020; Rupesh Agrawal, 2020). Although the FDA has recently approved other therapeutics for the treatment of COVID-19, such as the anti-viral remdesivir (FDA, 2020c), the World Health Organization (**WHO**) withdrew its approval a month later (World Health Organization, 2020). Clearly, there is an urgent need for *in vitro* screening tools to increase the speed and precision of pre-clinical evaluation of potential COVID-19 therapeutics so that clinical resources can be focused on the most promising candidates.

To address this need, in this study, we show the clinically predictive potential of a microphysiological system (**MPS**; also called organ-on-a-chip) for rapid screening of cardiac liabilities associated with repurposed drug polytherapy. We chose the heart as a tissue of interest because all drugs need to be screened for cardiac liabilities such as arrhythmias, tachycardia, QT prolongation, and Torsade’s de pointes (**TdP**), all of which can lead to sudden cardiac arrest. Furthermore, as recent studies have shown that SARS-CoV-2 infections can damage the heart’s contractile proteins (Perez-Bermejo et al., 2020), and cardiac comorbidities increase the likelihood of hospitalization and death (Nishiga et al., 2020; Sanyaolu et al., 2020; Ssentongo et al., 2020), there is an urgency for rapid drug screening tools predictive of clinical cardiology. Our cardiac MPS is a microfabricated fluidic system, that promotes human induced pluripotent stem cell-derived cardiomyocytes (**hiPSC-CMs**) to self-arrange into an organized, three-dimensional beating cardiac muscle fiber (Mathur et al., 2015). We further enhance the prognostic capability of our system by exposing the tissues to an in-house developed maturation media, leading to pharmacology outcomes more reminiscent of adult drug response (Huebsch et al., 2020). The drug response from the cardiac MPS can be further matured and analyzed *via* computational approaches (Tveito et al., 2018; Jæger et al., 2020a; Jæger et al., 2020b).

We chose HCQ and AZM as an example of polytherapy approved by the FDA for emergency use as treatment of COVID-19. We have previously used the heart muscle MPS platform in a mock *in vitro* clinical trial, successfully predicting clinical trial outcomes for HCQ and AZM polytherapy (Charrez et al., 2021). This chronic experiment was performed over 10 days to replicate clinical trial protocols. Our focus in this work was to conduct acute dose escalation studies of HCQ and AZM, and their polytherapy to demonstrate the platform’s ability to identify dosing regimens that alleviate cardiac liabilities. To address rapid cardiotoxic screening, a novel 4-plex version of our cardiac MPS was employed for drug dose escalation studies. We used multiple metrics to assess the pro-arrhythmic risks of HCQ and AZM polytherapy. We measured beat rate corrected action potential duration prolongation (**cAPD**) as a proxy for clinical QT prolongation, changes in the beat waveform (triangulation), action potential and calcium transient maximum upstroke velocity, and the occurrence of early after depolarizations (**EADs**) and delayed after depolarizations (**DADs**) as a function of dose, to identify the cardiac risk for each drug tested. Employing a voltage sensitive dye and hiPSC-CMs with a genetically encoded calcium reporter, we obtained precise APD and calcium transient outcomes of the cardiac muscle tissues as they were treated with HCQ and AZM.

We identified a QT increase in HCQ treated tissue, and its correlation to arrhythmic events, consistent with retrospective studies (Sharma et al., 2016; Blinova et al., 2017; Jankelson et al., 2020) and clinical literature (Asensio et al., 2020; Bessière et al., 2020). Our cardiac MPS showed no change in QT interval upon acute exposure to AZM, while observing a significant increase in arrhythmic events, indicating that the cardiac MPS predicted arrhythmias even in the absence of QT prolongation (Yang et al., 2017). Polytherapy showed a synergetic increase of QT interval when compared to monotherapy treatments. This observation was in line with literature data that reported a worsening of QT interval increase with the combination therapy (Chorin et al., 2020a; Chorin et al., 2020b; Bessière et al., 2020; Mercuro et al., 2020). Finally, proteomics analysis of effluent enabled the discovery of biomarkers that were directly correlated with cardiotoxicity and cellular damage. The outcomes of this paper correlate with published clinical results, validating our cardiac MPS as a good and rapid predictor of drug-induced arrhythmias.

## Materials and Methods

### Fabrication of Cardiac Microphysiological System

Microfluidic cardiac MPS were formed using an optimized version of the protocol described in our previous work (Mathur et al., 2015; Huebsch et al., 2020). Each MPS consisted of four multiplexed cell-loading port leading to their respective cell culture chambers (1300 μm × 130 μm) and two media ports, one media inlet and outlet per MPS. The media channels run adjacent to each side of the four cell chambers, separated by an array of fenestrations (2 µm × 2 µm high/width) to deliver nutrients while protecting the tissue from media flow shear stress. Two step photolithography was used to fabricate the microdevice silicon wafer mold, containing the features of multiple MPS. The first photolithography step was 2 µm high for the fenestration pattern: The piranha cleaned wafer was put onto a headway spinner where SU8 2002 (Kayaku Advanced Materials, Westborough, MA) was poured and spun (first 500 rpm for 10 s with 100 rpm/s acceleration; then 2000 rpm for 30 s with 300 rpm/s acceleration). The wafer was baked for 1 min at 95°C, exposed at 80 mJ/cm^2^ UV exposure (Karl Suss MA6 mask aligner with i-line) and baked again for 2 min at 95°C. Non-crosslinked photoresist was developed in SU8 developer for 1 min 15 s, and the wafer was hard baked at 180°C for 30–45 min. The second layer for cell chamber and media channel (55 μm high) could then be fabricated onto of the fenestration with a similar protocol. SU8 3050 (Kayaku Advanced Materials) was used and spun (first 500 rpm for 10 s with 100 rpm/s acceleration; then 3000 rpm for 30 s with 300 rpm/s acceleration). The wafer was baked slowly to allow for improved resolution: starting at 30°C, the temperature was increased to 65°C at a rate of 10°C/min. Once at 65°C, the temperature was kept for 10 min before ramping up to 95°C at a rate of 5°C/3 min. Wafers were kept at 95°C for 45 min. The wafer was then allowed to cool down slowly to room temperature before being exposed to UV at 160 mJ/cm^2^. Alignment marks allowed for perfect overlay of both layers. Post exposure bake was the same as previously described, with only 2 min at 65°C and 10 min at 95°C. Finally, wafers were developed for 35 min in shaker and sonicator before being hard baked at 180°C for 30–45 min. The cardiac MPS was formed by replica molding from the silicon wafer with polydimethylsiloxane (PDMS; Sylgard 184 kit, Dow Chemicals, Midland, MI) at a 10:1 ratio of Sylgard base to crosslinker. PDMS chips were then bonded to glass slides using oxygen plasma for 24 s, RF power 21W, and 100% oxygen flow.

### Fluid Dynamic Modeling

Microfluidic cardiac Characterization of fluid flow and wall shear stresses in the cardiac MPS was done *via* solving the Navier-Stokes equation using the finite element solver, COMSOL Multiphysics® (v 5.4, COMSOL, Inc. Burlington, MA). The computational domain was constructed by importing the geometry of the fluidic channels of the MPS. Fluid was modeled to be Newtonian and incompressible with a density of 1 g/mL and a dynamic viscosity of 1 mPa·s, and the flow within the fluidic channels were assumed to be laminar. Inlet boundary condition was set to mass-influx (based on the design flowrate of 30 μL/min) and outlet boundary condition was set to a gauge pressure of 0 Pa. For all other boundaries (i.e. walls), no-slip conditions were applied. Discretization of the computational domain was performed using the physics driven extra fine mesh. Steady state solver was used to obtain the velocity as well as the wall shear stress profiles within the MPS.

### Cell Source

Wild type C (WTC) hiPSC was used for this study. The cell line has a single-copy of CAG-driven GCaMP6f knocked into the first Exon of the AAVS1 “safe harbor” locus (Huebsch et al., 2015). It can be purchased from the Coriell Repository (# GM25256 hiPSC from fibroblast).

### Cardiomyocyte Differentiation

WTC hiPSC were differentiated to cardiomyocytes (CM) through small molecular manipulation of Wnt signaling pathway (Lian et al., 2013; Lian et al., 2014; Dunn and Palecek, 2018). Canonical Wnt ligands promote cell fate determination during embryonic development *via* inhibition of glycogen synthase kinase 3 (Gsk3) with CHIR99021. A decrease in Gsk3 expression leads to the nuclear accumulation of β-catenin and the subsequent activation of cardiac differentiation genes transcription machinery. To obtain fully differentiated CM, an inhibitor of Wnt (IWP4) is used to stop Wnt protein secretion and activity by preventing the palmitylation of Wnt proteins by Porcupine. Thawed hiPSCs were passaged three times at a density of 20,000 cells/cm^2^ in mTeSR-1 media (StemCell Technologies, Vancouver, Canada) with 10 µM Y27632 (Peprotech; Rocky Hill, NJ). After 3 days of expansion the differentiation was initiated. At day 0, the >90% confluent hiPSC wells were exposed to 8 µM CHIR99021 (Peprotech) in Roswell Park Memorial Institute (RPMI) 1640 medium containing B-27 supplement without insulin (RPMI-I). The next day, cells were fed with RPMI-I only. On day two, cells were exposed to 5 µM IWP-4 (Peprotech) and 150 μg/mL *L*-ascorbic acid (LAA) in RPMI-I for 2 days. On day four, cells were fed with RPMI-I only for two more days. From then onwards, RPMI containing standard B-27 supplement with insulin (RPMI + C) was added every second day. Typically, CM start beating on day eight of differentiation. After 15 days, hiPSC-CM were singularized with collagenase type II (Worthington Biochemical Corporation, Lakewood, NJ) for 45–60 min at 37°C and subsequently plated at a density of 100,000 cells/cm^2^ onto Matrigel, in EB20 with 10 µM Y27632 (new “day 0” after replating) (Huebsch et al., 2020). The next day, medium was exchanged for RPMI + C. At day three, cells were fed with RPMI 1640 [no glucose, with pyruvate supplemented with 23 mM sodium bicarbonate and 4 mM Sodium *L-*lacate (Tohyama et al., 2013)] for a total of 4 days (exchanged every other day) to allow for cardiomyocytes only to survive. At day seven, cells were washed with dPBS and fed with RPMI + C to recover. Two days after that, cardiomyocytes were singularized, and a fraction of them was characterized by flow cytometry for Cardiac Troponin T, and the remaining cells were loaded into cardiac MPS. Only differentiations with >95% cells positive for Cardiac Troponin T were subsequently loaded in the MPS.

### Loading of Cardiomyocytes and Microtissues Assembly Within Microphysiological System

Singularized- lactate-purified WTC hiPSC-derived cardiomyocytes were suspended in EB20 media supplemented with 10 µM Y27632. Density was calculated so that 3 µL of the cell suspension corresponded to 4,000 cells, that were injected into the loading port of each MPS. After 3 min centrifugation at 300 *g*, MPS were inspected under the microscope. Chambers that were not filled at this point were discarded. MPS were fed with 200 μL EB20 media supplemented with 10 µM Y27632 into the inlet tip and gravity would allow for a constant flow to the outlet until equilibrium was reached. The following day and every other day from then on, media was changed to our in-house “Maturation Media” (MM) as described in (Huebsch et al., 2020). MPS tissues were allowed to mature for 10 days before any subsequent experiments were performed.

### Pharmacology Studies

**FIGURE 1:**
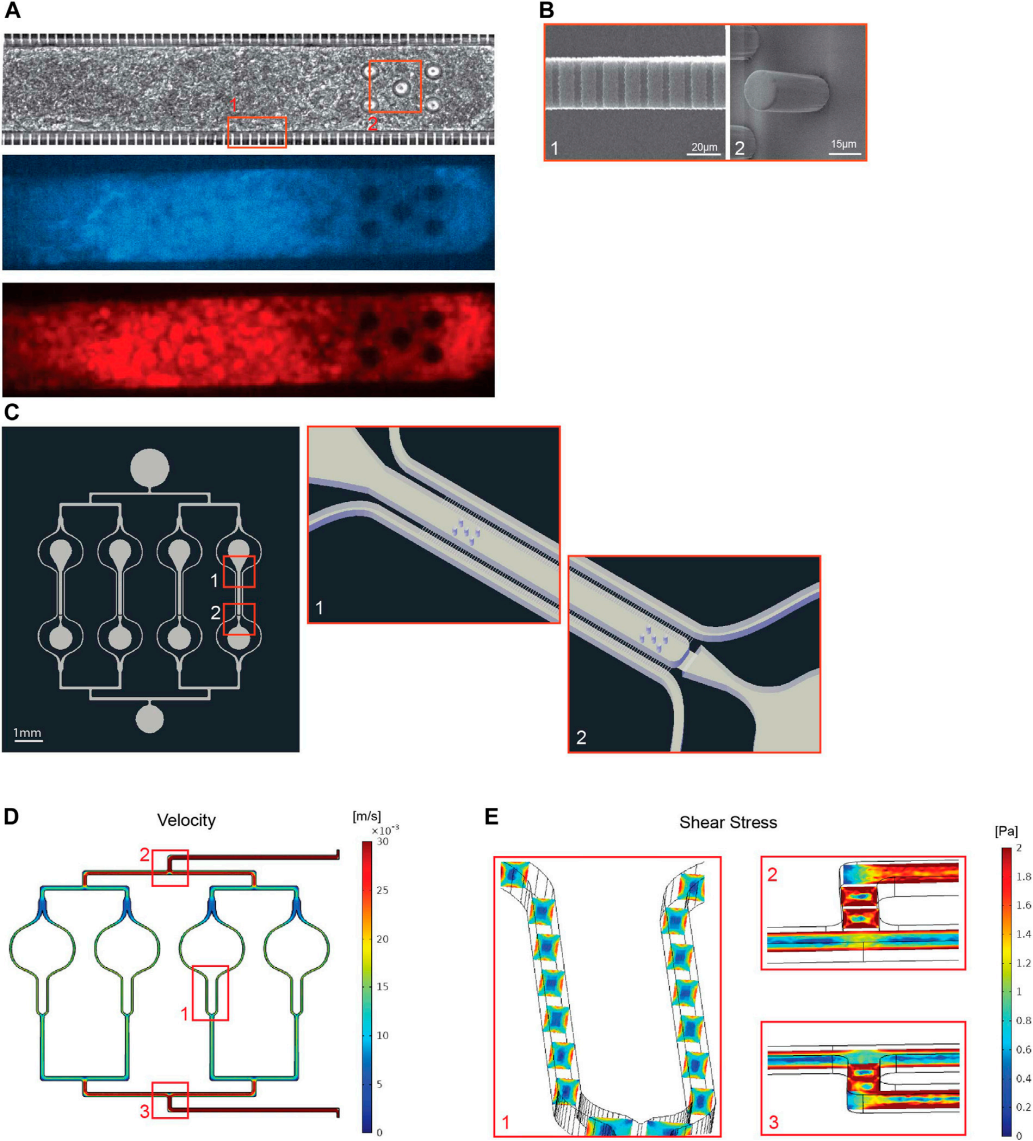
Design of the multiplexed cardiac microphysiological system (MPS). **(A)** 20x images of a cardiac MPS loaded with 4,000 human induced pluripotent stem cell derived cardiomyocytes. Top image shows brightfield capture of the tissue, middle image shows GFP fluorescence for calcium transient recordings, and bottom shows FarRed fluorescence of the voltage dye staining. **(B)** To left SEM image shows the fenestrations (2μm × 2μm x 20μm: h-w-l) required for protecting tissue in the cell chamber from media flow-induced shear stress (panel **E**) while enabling diffusion of nutrients. The right SEM image is a close up on pillars that keep the tissue fiber spanned along the length of the cell chamber. Both images correspond to area from panel (A) denoted by one and two respectively. **(C)** llustration of the 4-plex tissue chamber MPS (left) and a three dimensional schematic (right) of top (1) and bottom (2) portions of one tissue chamber, which dimensions were 1300μm × 130 μm with five 20 μm diameter pillars of at the top and the bottom of the chamber. **(D)** Illustration of the fluidics velocity profile obtained with COMSOL Multiphysics for volume flow rate of 30 μL/min, ranging from 30 mm/s at the inlet and dropping to 16 mm/s equally around each cell chamber. **(E)** Close up showing shear stress profiles in the media channels around the cell chamber (1), at the first (2) and last (3) bifurcation of the multiplex chip.

Twenty-four hours prior to any experiment, MPS tissues were stained with 500 nM of the action potential dye, BeRST-1, synthesized and quality controlled as described by Miller et al. (Huang et al., 2015). On the day of pharmacology study, drugs were weighed and dissolved in maturation media (**MM**) with 50 nM BeRST-1 voltage dye and adjusted vehicle concentration: all AZM doses had the same solvent conc. 0.5% EtOH. Hydroxychloroquine Sulfate (PHR1782-1G, Sigma, St. Louis, MO) was dissolved in PBS at 4.34 mg/mL and sterile filtered to create a 10 mM stock solution. The stock was always prepared fresh at the day of the experiment. Azithromycin (75199-25 MG-F, Sigma) was dissolved in 100% ethanol to create a 10 mg/mL (=13.35 mM) stock. The stock was stored at −20°C for up to 3 days. From the stock concentration, drugs were diluted to the highest dose tested and serial dilutions were made up to the lowest concentration range. Doses were chosen to vary around clinical C_max_ of HCQ at 1 μM (Yao et al., 2020) and AZM at 0.67 μM (Gautret et al., 2020) (Table 1). Once the drug solutions were freshly prepared, MPS were placed onto a heated plate (Tokai Hit, Gendoji-cho, Japan) at 37°C for 20 min to let them stabilize. Their action potential was recorded and only tissues with APD<500 ms were selected for study. Seven tissues per drug were used for the studies. A pressure driven pump system (Fluigent, N. Chelmsford, MA) comprising a 10-way selection valve (M-switch) and flow sensor (size M) was used for controlled and automated perfusion of increasing drug doses through the cardiac MPS. The flow sensor enabled a regular flow of 30 μL/min throughout the experiment. Supplementary Figure 1 for a schematic of the setup. When two microfluidic chips were hooked up in parallel, micrometering needle valves were included downstream of the MPS to adjust for equal flow into both sides. Each chip housed four tissue chambers (Figure 1). Once the tissues were selected based on their inclusion criteria (APD<500 ms and good visual quality), the fresh drug solutions were added to the Fluigent system. The tubing was primed with the different drug doses and the MPS inlet was connected to the Fluigent system outlet. The MPS outlet tubing was connected to an eppendorf for effluent collection. Alligator clips, connected to a Myopacer Field Simulator (IONOPTIX, Westwood, MA), were attached to the stainless-steel elbows (SC20/15 bent to 90°, Instech Laboratories, Plymouth Meeting, PA) of the MPS inlet and outlet to pace the tissues. Once the whole system was setup, with constant 30 μL/min flowrate through the MPS, the tissues were left for 30 min to stabilize. At this point, initial dose 0 recordings of spontaneous and paced activity were performed. The pump M-switch was then changing its output to the lowest drug dose, dose 0 effluent tube was replaced by an empty tube. MPS were incubated for 30 min. Spontaneous and paced activity was then recorded for the first dose and the process was iterated for increasing doses until all spontaneous and paced activity ceased or the highest dose was reached.

**TABLE 1:** Drug and doses used for both acute and chronic exposure.

Drug	Primary purpose	Clinical C_max_	Acute drug dose (*C_max_)
Hydroxychloroquine—HCQ (*PHR1782-1G, Sigma*)	Anti-malarial	1 μM Yao et al. (2020)	0, 0.1, 1, 10, 100, 1000
Azithromycin—AZM (*75,199-25 MG-F, Sigma*)	Macrolide antibiotic	0.67 μM Gautret et al. (2020)	0, 0.1, 1, 10, 100
HCQ + AZM combination	SARS-CoV2	—	**HCQ**: 0, 1, 1, 1, 1, 1, 1
			**AZM**: 0, 0, 0.1, 0.3, 1, 3, 10

### Image Acquisition for Pharmacology Studies

Initial spontaneous recordings were the following: 6- and 30-s videos of fluorescent calcium flux as well as 6-s voltage dye videos. After that, the tissues were field paced at 1 or 1.5 Hz. Since the multiplex design has four tissue for one media inlet and outlet, all tissues were paced at the same frequency. Tissues were paced at 1 Hz, and if at least one of the four tissues did not capture the 1 Hz pacing the frequency was increased to 1.5 Hz. The same frequency was kept for the entire drug dose escalation study. For each recording, tissues were paced for 10 s (3 V, 20 msec bipolar pulses) *via* stainless steel elbow fluidic connectors inserted in both the media inlet and outlet. Pacing was maintained while 6 s calcium and voltage videos were acquired. Once spontaneous and paced recordings were performed, MPS were exposed to higher drug concentration, incubated for 30 min before the next recording sequence was started. A NIKON TE300HEM microscope with a HAMAMATSU digital CMOS camera C11440/ORCA-Flash 4.0 was used and enabled 100 frames per second videos. For fluorescence imaging, a Lumencor (Beaverton, OR) SpectraX Light Engine and filtered with a QUAD filter (Semrock (IDEX), Rochester, NY) was used. GCaMP videos used the Cyan LED, 470 nm (4 × 4 binning, 10 ms), while BeRST-1 videos used the Far-red LED, 640 ms (4 × 4 binning, 10 ms). Videos were acquired using Nikons NIS-Elements software. Post-experiment processing was performed with an in-house developed Python library that could analyze the fluorescence intensity over the time of the recording.

### Thorough Electrophysiology Analysis as a Proxy for Clinical QT Interval Study and Arrhythmia Prediction

Clinically, QT interval increase is often associated with arrhythmias, such as Torsade de Pointes (**TdP**) (Hondeghem et al., 2001). TdP is a ventricular tachycardia triggered by Early afterdepolarizations (**EADs**), where the amplitude and configuration of ECG measurements vary continuously. EADs represent a transition from slowed cellular repolarization to unstable (chaotic) repolarization at the cellular level. (Sato et al., 2009; Qu et al., 2013). Patients with QT interval >500 ms are considered at risk for TdP (Asensio et al., 2020), therefore all tissues with baseline APD >500 ms were excluded from the study. In this study, we used rate corrected action potential duration at 80% of its repolarization (APD_80_) as a proxy for QT interval measurements (Blinova et al., 2017). Rate correction was performed with the Fridericia correction (Vandenberk et al., 2016), or by using data from paced tissues. Hondeghem et al. (2001) showed that if APD prolongation was accompanied by instability or triangulation then, EADs and proarrhythmia were more certain to follow. In this study, we defined triangulation as (APD_80_—APD_30_) normalized to APD_80._ We also used Poincaré plots to study the instances of instabilities, where the *n* ^th^ APD_80_ is plotted against the (n-1)^th^ APD_80_, each normalized to the mean APD_80_. In the case of stable APD_80_ in successive beats, overlaying traces at the origin of the graph will be visible (Hondeghem et al., 2001), appearing as a asymmetric circle. However, if there are large deviations between successive APD, large disorganized polygons will be observed that correlate with cardiac arrhythmias. We also characterized arrhythmic instances qualitatively by studying 30 s long calcium traces. EAD, DAD or change in amplitude and shape (loss of plateau phase—typical TdP trait) were considered abnormal ECG and arrhythmic events. If the maximal amplitude of a trace was less than 60% the one of control data, or if no beating was observed, the datapoint was qualified as weak or no beating signal.

Both the action potential and calcium transient maximum upstroke velocity, corresponding to the maximum of the upstroke’s slope (max derivative—max dF/dt), is calculated with our in-house Python software.

### Plasma Protein Profiling Using Olink Multiplex Panel

All effluent samples were kept at −80°C until sent on dry ice and shipped to Olink Proteomics (Watertown, MA) for protein quantification. Olink Proteomics uses multiplex proximity extension assay (PEA) panels (Assarsson et al., 2014), where two matched antibodies labeled with unique DNA oligonucleotides simultaneously bind to a target protein in solution. Their DNA oligonucleotides will hybridize into a double-stranded DNA “barcode” which is unique for the specific antigen and quantitatively proportional to the initial concentration of target protein. The hybridization and extension are followed by PCR amplification and the amplicon is then finally quantified by microfluidic qPCR using Fluidigm BioMark HD system (Fluidigm Corporation, South San Francisco, CA). The Organ Damage panel (https://www.olink.com/products/organ-damage-panel/) was used in this study. It consists of 92 unique markers of toxicity and cellular damage. In the data post processing, we only included experiments with >65% of the samples above the limit of detection. The limit of detection is set at 3 standard deviation above negative control values.

### Scanning Electron Microscopy

Freshly fabricated PDMS MPS were sputtered with gold/palladium (AuPd) for 60 s at 10 mA to get a 10 nm conductive layer at the surface (Cressington sputter coater 108 auto, Watford, England, United Kingdom). The sample was then imaged in a scanning electron microscope (Quantum 3D FEG, Orion Nanofab, ZEISS, Oberkochen, Germany) with the following settings: 30 μm aperture, 20 kV, 120 pA, ×40° tilt, ×1974 magnification, 30 μs scan rate.

### Statistics

All statistics were calculated using GraphPad Prism (San Diego, CA). All electrophysiology graphs statistics were analyzed with one-way ANOVA with Dunnett’s post-hoc analysis and multiple comparison to dose 0 for HCQ and AZM. Multiple comparison to no drug and HCQ only was run for the AZM + HCQ combination acute drug study. One way ANOVA, multiple comparison to dose 0 and to one another with Dunnett’s post-hoc analysis was also run on the Olink proteomics data. Non-parametric Chi-squared approximation was run for qualitative arrhythmic events assessment in a pairwise manner. Significance was determined with *p*-value < 0.05. *p < 0.05; **p < 0.01; ***p < 0.001, ****p < 0.0001.

## Results

### Multiplexing of Cardiac Microphysiological System and Fluid Dynamic Modeling

We optimized our previously published cardiac MPS (Mathur et al., 2015) in a number of ways, including chamber design, cell composition, multiplexing and pressure driven fluidic pump. Cardiomyocyte function was optically recorded prior and during experiments with both voltage (BERST-1 voltage sensitive dye) and calcium transient (GCAMP encoded calcium sensor) reporters (Figure 1A). We added five mechanosensing pillars of 20 μm diameter (Figure 1B, right) at the top and the bottom of the cell chamber to act as a resistance against which the tissue will exercise for enhanced tissue development and contraction force. The cell chamber dimensions were 1300 μm × 130 μm (Figure 1C). Absorption studies showed no significant drug binding to the microfluidic system for HCQ or AZM at any dose (Supplementary Figures 2A,B). These findings confirmed that the MPS were indeed exposed to intended concentrations. The free fraction of drug in our culture media was almost 90% for HCQ and 75% for AZM (Supplementary Figure 2C). Fluid mechanical characterization of the cardiac MPS was done *via* solving the Navier-Stokes equation using the finite element solver, COMSOL Multiphysics® (Burlington, MA, United States) to obtain the velocity (Figure 1D) as well as the wall shear stress (Figure 1E) profiles within the device. Our analyses show that the maximum values of velocity and wall shear stress within the MPS are 16 mm/s and 1.6 Pa ([6 dyn/cm^2^, within the range of blood vessel wall shear stress (Koutsiaris et al., 2007)], respectively. Furthermore, the simulations confirmed that the cardiac tissue is protected from the media shear stress by the endothelial-like fenestration barrier (Mathur et al., 2015). The protective barrier also allows for rapid delivery of small molecules to the tissue, supplying a sufficient amount of nutrients throughout the study, as previously described (Mathur et al., 2015).

### Acute Exposure to Hydroxychloroquine Triggers QT Prolongation and Rhythm Instability, Leading to Arrhythmic Events and Stop of Spontaneous Beating Activity

**FIGURE 2:**
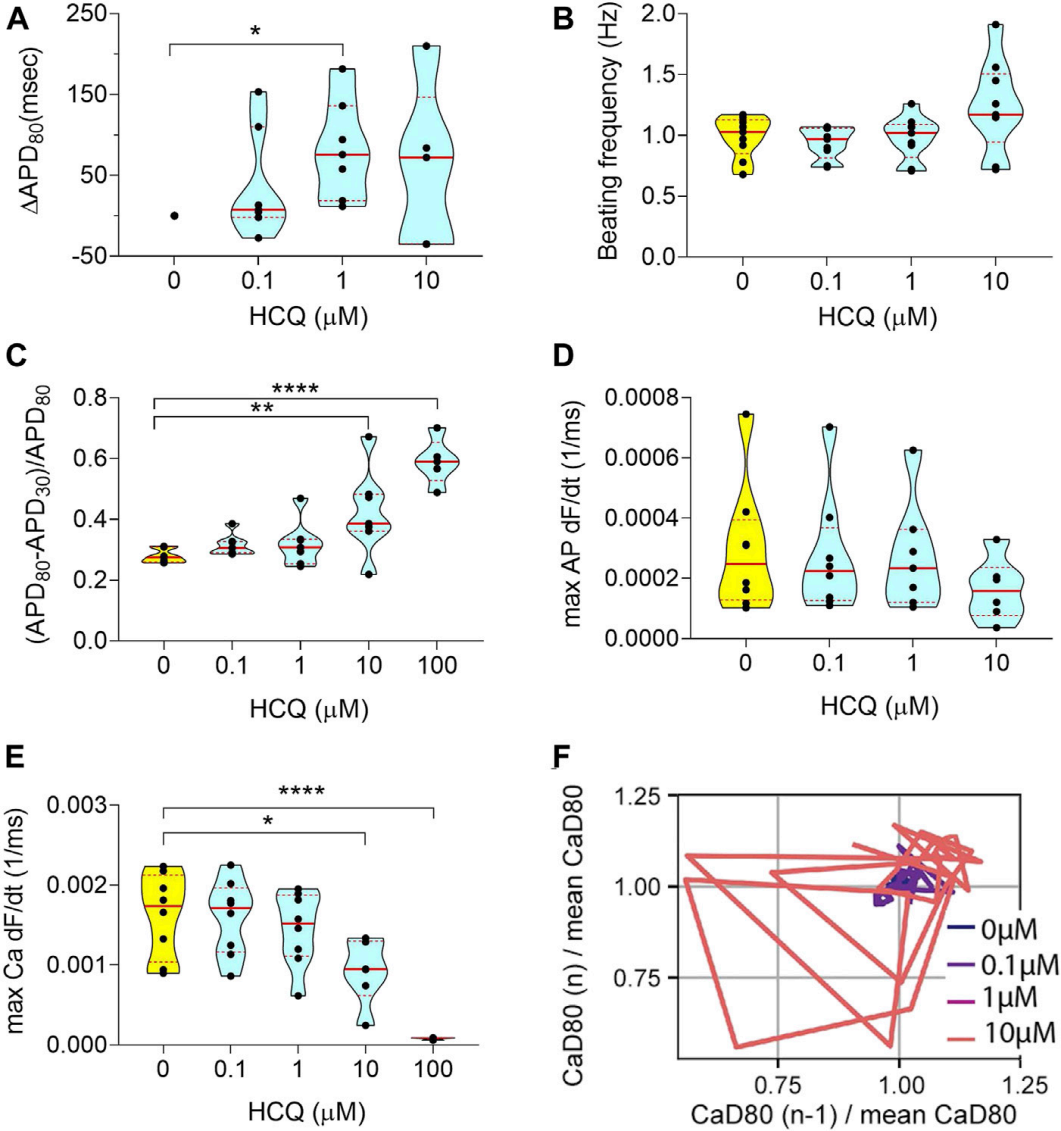
Electrophysiology analysis of acute HCQ exposure. **(A)** Violin plot depicting the change in APD_80_ in paced tissues over increasing doses (100 μM was not shown, due to poor signal quality). Values were normalized to baseline 0 μM of each tissue chamber. **(B)** Graph showing spontaneous beating frequency in Hz, for up to 10 μM HCQ. **(C)** Triangulation values of spontaneous action potential traces. **(D)** Violin plot showing the action potential maximum upstroke velocity, calculated as the maximum of the upstroke slope of paced traces. The 100 μM data were not shown due to poor signal quality. **(E)** Graph describing the maximum upstroke velocity of paced calcium transient traces. **(F)** Representative Poincaré plot showing instabilities in calcium transient duration in response to increasing HCQ doses (data from spontaneous traces). All tissues analyzed were within inclusion criteria of APD_80_ < 500 ms at baseline. Statistics run were one-way ANOVA with multiple comparison to baseline 0 μM and Dunnett’s post-hoc correction. *p < 0.05; **p < 0.01; ****p < 0.0001.

**FIGURE 3:**
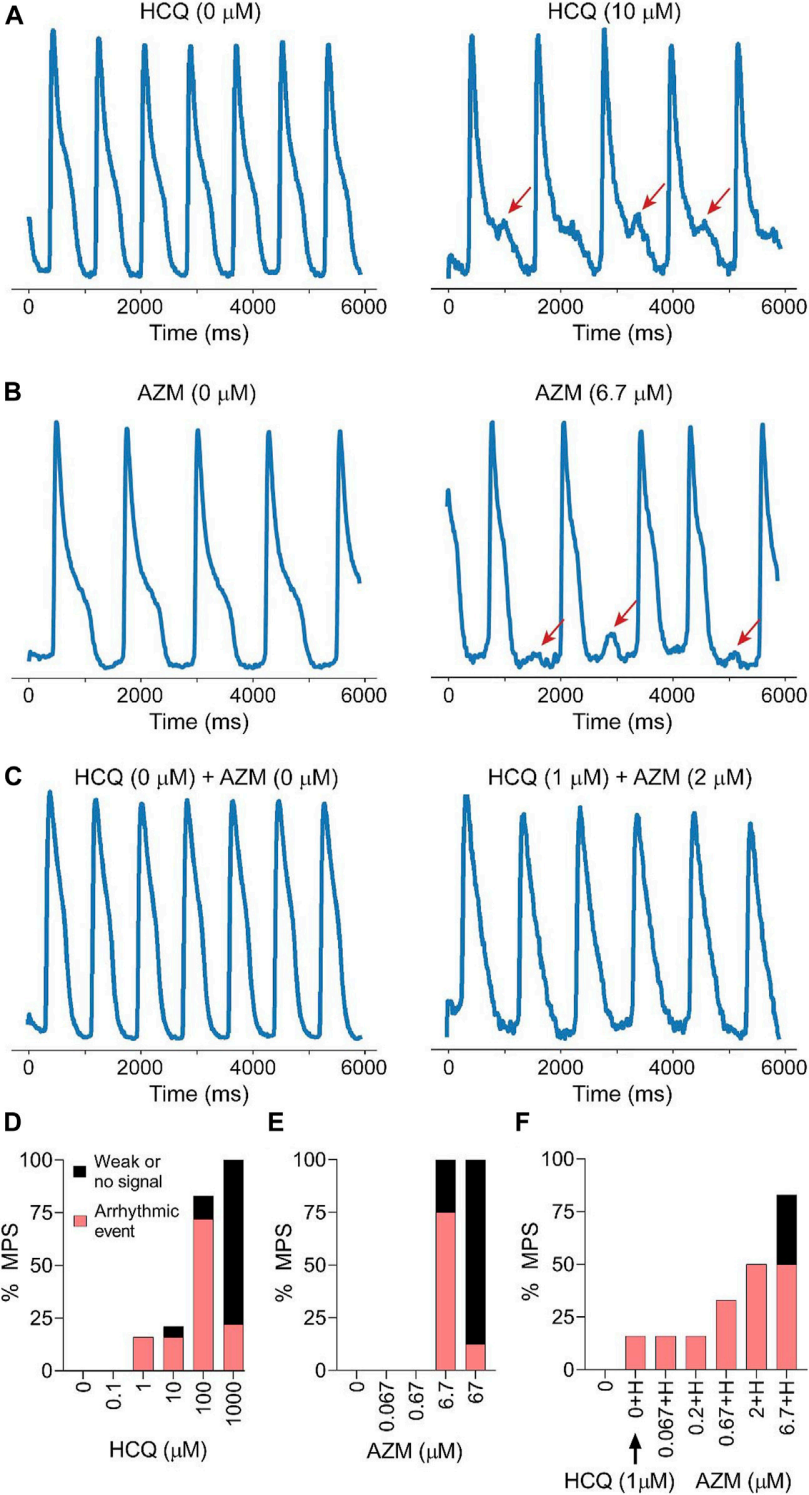
Drug induced arrhythmia for acute HCQ, AZM, and polytherapy. **(A)** Representative calcium transient trace at baseline **(left)** and 10 μM **(right)** HCQ, where early after-depolarization (EAD) can be observed (red arrows). **(B)** Representative calcium transient trace at baseline **(left)** and 6.7 μM **(right)** AZM, where delayed after-depolarization (DAD) can be observed (red arrows). **(C)** Representative calcium transient trace at baseline **(left)** and 1 μM HCQ combined with 2 μM AZM **(right)**, where calcium trace amplitude and configuration change. **(D,E,F)** Histogram showing incidence of arrhythmia-like events such as EAD, DAD or irregular beat shape (red) and weak signal or beating cecassion (black) as percentage of total MPS for HCQ only **(D)**, AZM only **(E)** and combination therapy **(F)**. Traces used were spontaneous 30 s recording of calcium traces.

We performed a dose-escalation study for HCQ using the cardiac MPS. It is known that HCQ inhibits *I*_*Kr*_ potassium channels (also named *hERG* channels) (Jankelson et al., 2020), triggering an increase in QT interval of cardiomyocytes. QT interval above 500 ms is typically correlated with arrhythmias that are responsible for sudden death (Jankelson et al., 2020). We observed an increase of APD_80_ at the clinical C_max_ of 1 μM as well as an increase in the AP triangulation after 10 μM (Figures 2A,C). The beat frequency did not change with increasing HCQ dose (Figure 2B). The voltage upstroke velocity decreased at 10 μM, although not significantly (Figure 2D), while the calcium upstroke velocity showed a dramatic decrease with HCQ increasing dose, especially at 10 and 100 μM (Figure 2E). Rhythm instabilities were also observed in Poincaré plots for 67% of the tissues at 10 μM (Figure 2F). Interestingly, APD and triangulation increases were correlated with EADs (calcium traces) (Figure 3A). We observed arrhythmic events in 72% of the tissues at 100 μM and 22% at 10 μM, and 78% of tissues showed weak or no beating signal at 1000 μM (Figure 3D).

### Acute Exposure to Azithromycin Triggers Rhythm Instability, Correlated to Arrhythmic Events and Stop of Spontaneous Beating Activity

**FIGURE 4:**
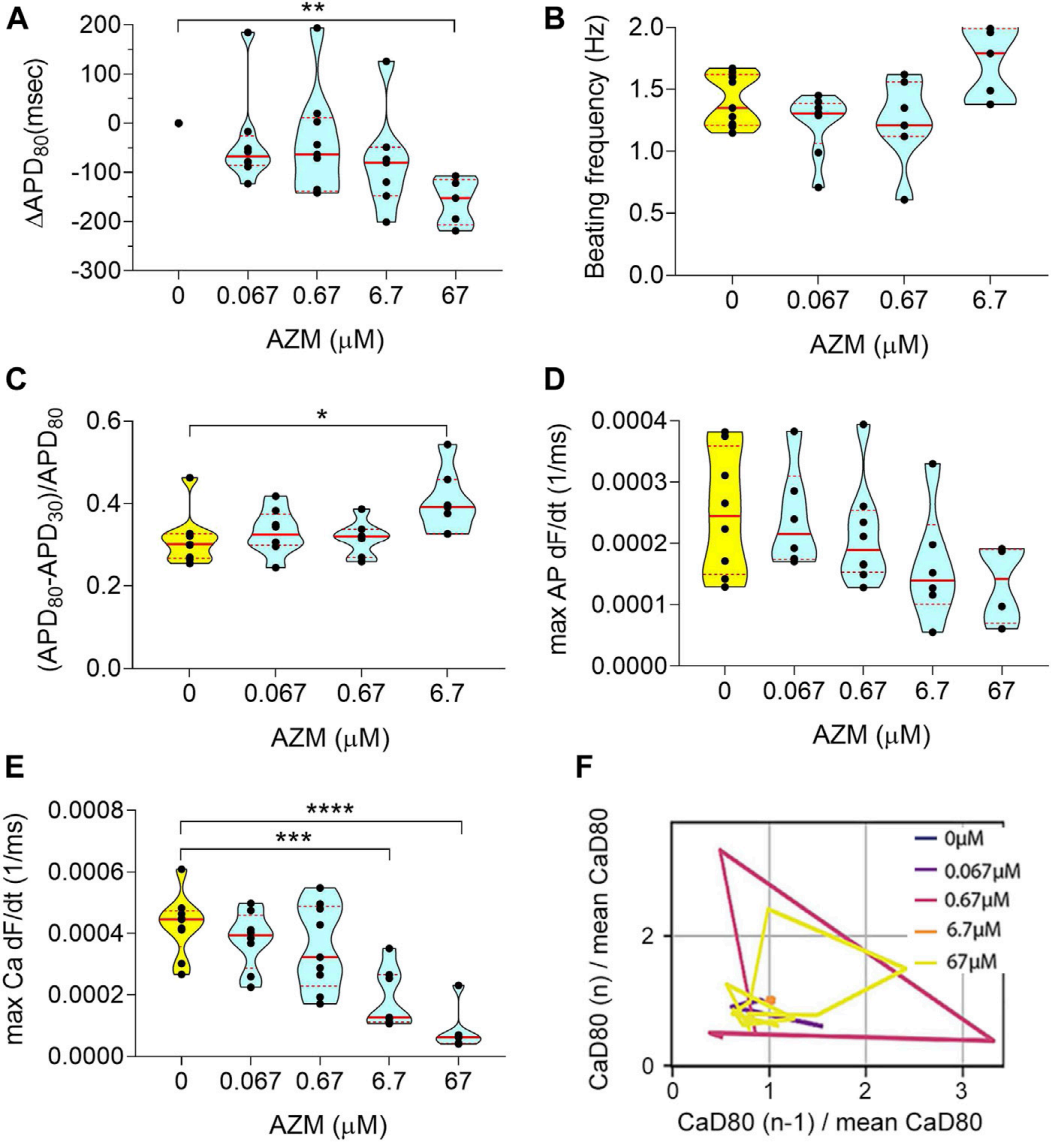
Electrophysiology analysis of acute AZM exposure. **(A)** Violin plot depicting the change in APD_80_ in paced tissues over increasing doses. Values were normalized to baseline 0 μM of each tissue chamber. **(B)** Graph showing spontaneous beating frequency in Hz, for up to 6.7 μM AZM. At 67 μM tissues stopped beating spontaneouly and data was therefore excluded from the graph. **(C)** Change in triangulation of spontaneous action potential traces. The 67 μM data was not shown, due to cease of spontaneous beating activity. **(D)** Violin plot showing the action potential maximum upstroke velocity, calculated as the maximum of the upstroke slope of paced traces. **(E)** Graph describing the maximum upstroke velocity of paced calcium transient traces. **(F)** Representative Poincaré plot showing instabilities in calcium transient duration in response to increasing AZM doses (data from spontaneous traces). All tissues analyzed were within inclusion criteria of APD_80_ < 500 ms at baseline. Statistics run were one-way ANOVA with multiple comparison to baseline 0 μM and Dunnett’s post-hoc correction. *p < 0.05; **p < 0.01; ***p < 0.001, ****p < 0.0001.

We performed a dose-escalation study for AZM using the cardiac MPS. AZM is also associated with an increased risk of cardiac arrhythmia (Ray et al., 2012), although not necessarily related to QT increase (Yang et al., 2017; Roy and MainakMukhopadhyay, 2020). The APD_80_ did not show any change up to 6.7 μM (10 × C_max_) and decreased at 67 μM (Figure 4A). However, at 67 μM, only 12% of AZM-treated tissues were beating spontaneously (Figure 3E), and therefore, the decrease in APD_80_ was most likely due to toxicity. We observed an increase in AP triangulation at 6.7 μM (Figure 4C). No significant change was seen in beating frequency (Figure 4B) or AP upstroke velocity (Figure 4D). Calcium transient upstroke velocity, however, showed a significant decrease starting at 6.7 μM (Figure 4E). We observed an increased rhythm instability starting at 0.67 μM (C_max_) to 6.7 μM, where 60% of the tissues were unstable (Figure 4F). A typical arrhythmic observation in AZM arrhythmic MPS were delayed after depolarization (DAD, Figure 3B). Figure 3E shows that arrhythmic events occur in 75% of the tissues at 6.7 μM and by 67 μM 12% of tissues showed arrhythmias, while the remaining tissues had weak or no beating signal.

### Acute Exposure of Hydroxychloroquine and Azithromycin Polytherapy Triggers Triangulation and Rhythm Instability, Which Correlated With Arrhythmic Events

**FIGURE 5:**
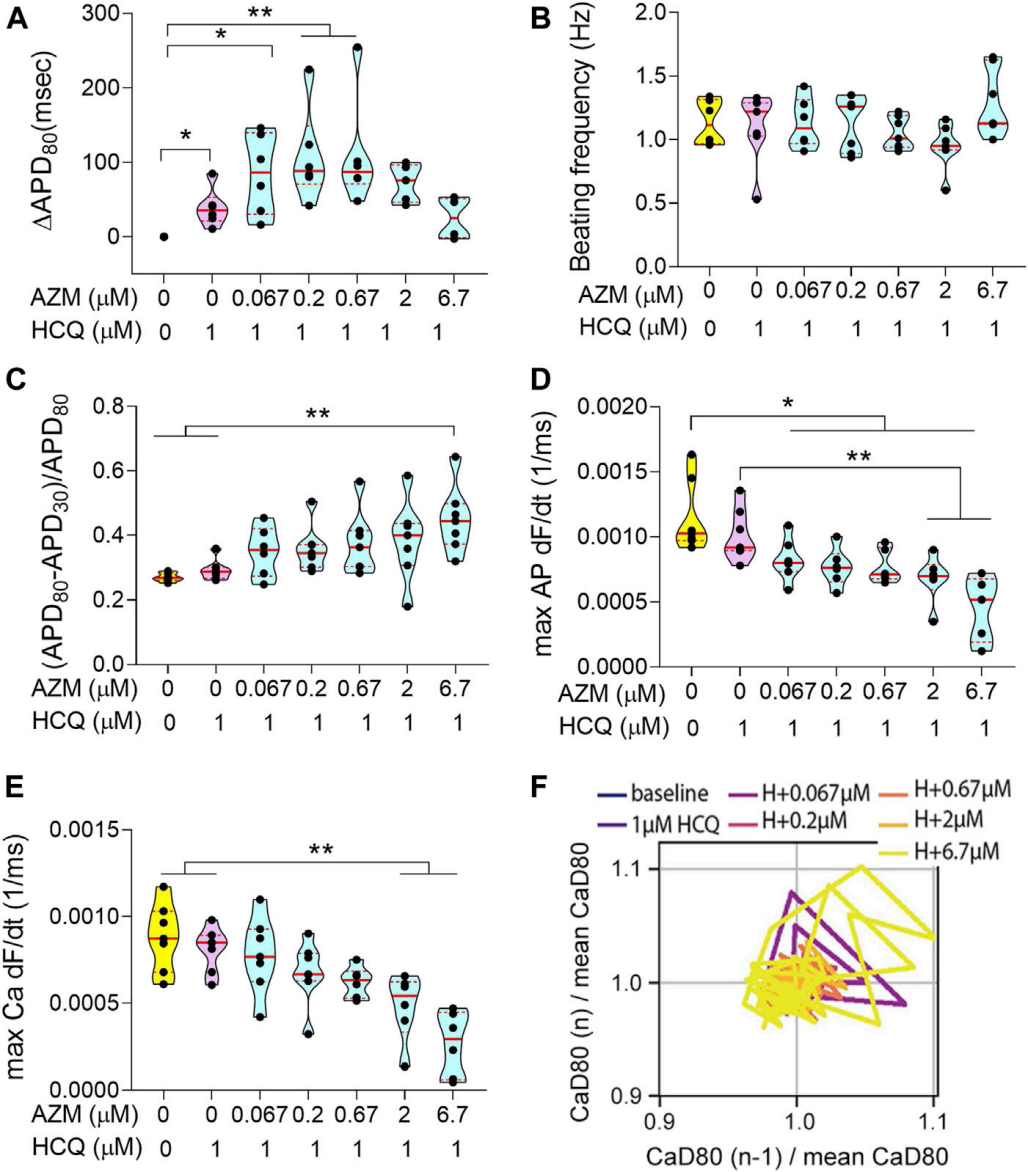
Electrophysiology analysis of acute combination of HCQ and AZM exposure. **(A)** Violin plot depicting the change in APD_80_ in paced tissues over increasing doses. Values were normalized to baseline 0 μM of each respective tissue. **(B)** Graph showing spontaneous beating frequency in Hz. **(C)** Triangulation values of spontaneous action potential traces. **(D)** Violin plot showing the action potential maximum upstroke velocity, calculated as the maximum of the upstroke slope of paced traces. **(E)** Graph describing the maximum upstroke velocity of paced calcium transient traces. **(F)** Representative Poincaré plot showing instabilities in calcium transient duration in response to HCQ + AZM polytherapy (data from spontaneous traces). All tissues analyzed were within inclusion criteria of APD_80_ < 500 ms at baseline. Statistics run were one-way ANOVA with multiple comparison to baseline 0 and 1 μM HCQ and Dunnett’s post-hoc analysis. Unpaired *t*-test comparison with Welch’s correction between 0 and 1 μM HCQ were performed to confirm observations seen in Figure 2, *t*-test only showed significance for A. *p < 0.05; **p < 0.01; ***p < 0.001,***p < 0.0001.

As observed in HCQ alone data (Figure 2A), the addition of HCQ at C_max_ (1 μM) increased the APD_80_ significantly (Figure 5A). Increasing concentrations of AZM were applied in combination with 1 μM HCQ and further increased the APD_80_ up to the AZM C_max_ (0.67 μM). At HCQ +6.7 μM AZM the APD_80_ reverted to the baseline value (Figure 5A), probably due to toxicity, as voltage signals became weak with arrhythmic events (Figure 3F). No change was observed in beat frequency (Figure 5B). The AP triangulation did not show a significant increase with 1 μM HCQ alone; however, the addition of AZM increased it continuously until a significant change was observed at the highest dose (Figure 5C). The combination of both drugs decreased the upstroke velocity for both AP (Figure 5D) and calcium (Figure 5E). Poincaré plots showed first occurrences of instabilities with the addition of C_max_ HCQ (16% of MPS). The incidence of instabilities increased further with AZM concentrations until 70% of MPS showed instabilities at 0.67 μM AZM. (Figure 5F). APD lengthening accompanied with triangulation increase, also increased rhythm instabilities and triggered arrhythmic events. Interestingly, most arrhythmic outcomes were TdP-like events (Figure 3C), where traces changed shape and amplitude (not shown since the traces’ amplitudes were normalized here), the plateau phase was absent and the signal became weak when compared to control traces. TdP are typically observed on ECG when traces continuously change configuration and amplitude (Asensio et al., 2020). We observed the appearance of arrhythmic events in 16% of the tissues starting with HCQ only and increasing to 33% with the addition of 0.67 μM AZM and 50% with 2 and 6.7 μM AZM. At the highest concentration, 30% of tissues showed weak or no beating signals (Figure 3F).

### Proteomics Analysis of Microphysiological System Effluent Reveals Cardiotoxicity Biomarkers to be Potentially Monitored in Patients Treated With Hydroxychloroquine and/or Azithromycin

**FIGURE 6:**
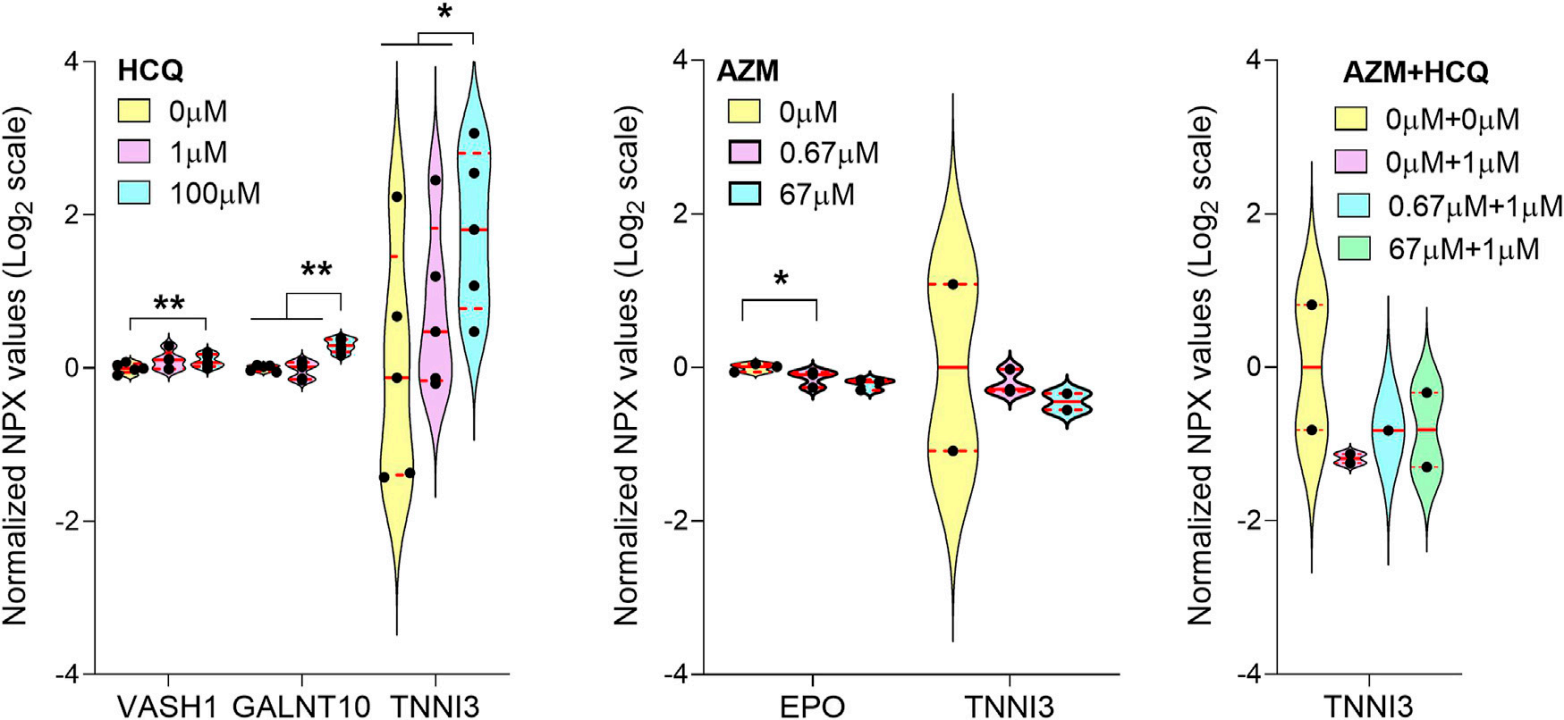
Proteomics Analysis of MPS mono and polytherpay. Violin plot with biomarkers showing significant changes with increasing doses of HCQ alone **(A)**, AZM alone **(B)** or polytherapy dose escalation **(C)**. Only biomarkers with >65% of samples above limit of detection were included. The limit of detection was set at 3 standard deviation above negative control values. Statistics run were one-way ANOVA with multiple comparison and Dunnett’s post-hoc correction. *p < 0.05; **p < 0.01.

We analyzed the effluent from the cardiac MPS microfluidic channels for 92 biomarkers and report only biomarkers related to cardiotoxicity that changed significantly upon acute exposure to either HCQ or AZM alone and their combination. We observed a significant increase of VASH1 and GALNT10 (Figure 6A) at 100 μM HCQ when compared to 0 μM. Cardiac troponin I, TNNI3, a well-known biomarker of cardiac injury, showed a significant increase at 100 μM when compared to both 0 and 1 μM HCQ (Figure 6A). Acute AZM exposure showed a significant decrease in EPO (Erythropoietine) (Figure 6B) at 67 μM when compared to 0 μM. No biomarkers changed significantly in the highest dose of polytherapy (1 × C_max_ HCQ +10 × C_max_ AZM) compared to no drug or 1 × C_max_ HCQ alone. No change in TNNI3 was observed for both AZM and polytherapy (Figures 6B,C), suggesting that high HCQ doses are more cardiotoxic than high doses of AZM alone or in combination with HCQ at C_max_.

## Discussion

Maximal AP upstroke velocity is a common measure of *I*_*Na*_ (Casini et al., 2009), and is an important metric to assess safety features of drugs; its dysfunction may cause life-threatening cardiac arrhythmias by reducing cardiac excitability (Tan et al., 2001; Chatre et al., 2018). Neither HCQ (Figure 2D) or AZM (Figure 4D) monotherapies showed a significant change in cardiac *I*_*Na*_. However, the polytherapy showed a synergistic decrease in the AP upstroke velocity when compared to monotherapies (Figure 5D). Interestingly, HCQ is a known *I*_*Na*_ blocker (Chatre et al., 2018) whereas AZM has been reported to potentiate cardiac *I*_*Na*_ (Yang et al., 2017), suggesting that HCQ-dependent block of *I*_*Na*_ dominates the effect of AZM on *I*_*Na*_.

Maximal calcium upstroke velocity is directly correlated to the speed of sarcoplasmic reticulum Ca^2+^ release, executed by ryanodine receptors opening (RyR) (Jaimes et al., 2016). HCQ has been shown to facilitate the oxidation and dysfunction of RyR (Zhang et al., 2020), which negatively impacts calcium upstroke velocity. HCQ is also an *I*_*CaL*_ blocker (Capel et al., 2015), which will decrease both calcium transient upstroke velocity and amplitude (Grantham and Cannell, 1996). We identified a decrease in calcium upstroke velocity with HCQ acute exposure (Figure 2E). AZM-dependent *I*_*Na*_ potentiation can also promote intracellular sodium loading, and subsequently dysregulates intracellular calcium concentration, that will lead to DAD (Antzelevitch and Burashnikov, 2011) (Figure 3B), and a decrease in calcium transient upstroke velocity (Figure 4E). The polytherapy synergistically decreased the calcium transient upstroke velocity (Figure 5E), suggesting that and AZM-dependent *I*_*Na*_ potentiation is required in addition to 1 μM HCQ to significantly decrease calcium upstroke velocity.

Increases in APD and triangulation, accompanied by instabilities (i.e., Poincaré plots), typically leads to arrhythmias (Hondeghem et al., 2001). HCQ has a known *I*_*Kr*_ block effect associated with QT prolongation and arrhythmic events (Sharma et al., 2016; Blinova et al., 2017; Jankelson et al., 2020). Our experimental action potential data are consistent with the clinical literature (Asensio et al., 2020; Bessière et al., 2020) and indicate that the cardiac MPS is a good predictor of the clinical cardiotoxicity of HCQ. Interestingly AZM has been associated with an increase in cardiovascular death, mostly through QT prolongation and arrhythmia (Ray et al., 2012; Vouri et al., 2021). However, it has also been shown that AZM lacks strong pharmacodynamic evidence of *I*_*Kr*_ inhibition, and that it can cause ventricular tachycardia in the absence of QT prolongation, indicating a novel proarrhythmic syndrome (Yang et al., 2017). This novel syndrome was successfully observed in our cardiac MPS: unchanged APD_80_ up to 6.7 μM AZM, with increased instabilities and arrhythmic events. The AZM data clearly show that the cardiac MPS can predict arrhythmias through beat shape changes (triangulation increase) and instabilities, even in the absence of APD_80_ prolongation. The latter is important for critical arrhythmia prediction and has been a focus of revised International Conference on Harmonization (IHC) E14 recommendations by both the CiPA initiative (Dutta et al., 2017a; Dutta et al., 2017b) and the pharmaceutical industry. The E14 guidance is intended to encourage the assessment of drug effects on the QT/QTc interval as a standard part of drug safety evaluation (Food and Drug Administrat, 2005).

The polytherapy study showed a significant increase of APD_80_, triangulation and instability as AZM dose was escalated in presence of a constant HCQ concentration, supporting literature that observed a worsening of QT interval increase with combination therapy (Chorin et al., 2020a; Chorin et al., 2020b; Bessière et al., 2020; Mercuro et al., 2020). In polytherapy we observed arrhythmia in 30–50% of the tissues at C_max_ HCQ + C_max_ AZM, corresponding to clinical data where 75% of patients who were given a combination of HCQ and AZM had ventricular arrhythmias (Gérard et al., 2020). Despite the presence of arrhythmic behavior, we did not see a significant change in arrhythmia instances when comparing C_max_ HCQ to C_max_ HCQ + AZM in polytherapy recordings. The latter suggests no worsening of arrhythmic behavior when AZM was added to C_max_ HCQ. This is in concordance with clinical data, concluding that there were no significant differences in the relative likelihood of abnormal ECG findings for polytherapy compared to monotherapy (Rosenberg et al., 2020).

The biomarker study showed an increase in numerous cardiotoxicity-related proteins when tissues were exposed to HCQ (Figure 6A). The marker VASH1 is associated with stiffer cardiac tissues and slower relaxation of failing human cardiomyocytes (Chen et al., 2020). GALNT10 has a critical role in protein folding, secretion and stability. Its upregulation has been shown to be a reparative response in mice 5 days post myocardial infarction (Hunt et al., 2012). Finally, a significant increase in cardiac troponin I (TNNI3), a well-known biomarker of cardiac injury (Sandoval et al., 2020; Lindner et al., 2020), correlates well with our observations of cardiotoxic effects of HCQ as well as clinically reported HCQ-related troponin I increase (Joyce et al., 2013). AZM exposure significantly decreased EPO (Figure 6B), which is indicative of cardiotoxicity (Santhanam et al., 2010). In cardiomyocytes, GALNT10 is typically an intracellular protein, suggesting cellular damage of the tissues at high doses of HCQ. No biomarkers changed significantly in the highest dose of polytherapy (C_max_ HCQ + 10 × C_max_ AZM) compared to no drug or C_max_ HCQ alone (Figure 6C). This matches with results of single drug experiments, where significant changes were only observed for very high doses (100 × C_max_) of the respective drug. Additional analysis will be needed to identify more subtle biomarker changes at lower drug concentrations.

In the 12 months following the official declaration of global pandemic in March 2020, emergency use authorization of drugs like HCQ and AZM have been granted and revoked, due to inconclusive efficacy of the drugs to reduce viral load. In 250 clinical trials that were started, with significant costs and resources of the healthcare system, many studies had inconclusive efficacy outcomes or were terminated due to cardiac liability issues. If used prospectively, our MPS would have successfully predicted the clinical cardiology response (Chorin et al., 2020a; Asensio et al., 2020; Chorin et al., 2020b; Bessière et al., 2020; Mercuro et al., 2020), in that the combination of both drugs would synergistically increase QT interval when compared to single drug exposure, while not impacting the overall frequency of arrhythmic events. Furthermore, by combining the cardiac MPS with *in silico* methods we can address mechanism of action, at an ion channel level, of potential drug side effects and better predict drug effectiveness (Tveito et al., 2018; Jæger et al., 2020b; Jæger et al., 2021). Thus, employing cardiac MPS prior to clinical trials would enable rapid evaluation of the cardiac safety of promising drug candidates and drug combinations.

## Conclusion

Our cardiac MPS, containing a 3D human heart muscle, can predict arrhythmic risk based on the observation of triangulation changes and instabilities, even if such arrhythmias occur in the absence of action potential duration prolongation. The methodology of this study can be extended to novel more promising therapeutics to treat COVID-19, and to MPS representing a disease state of the cardiac muscle. The high content, controlled and inexpensive MPS should help to rapidly evaluate safety properties of potential therapeutics in crisis times, and accelerate clinical decision making.

## Data Availability

The raw data supporting the conclusion of this article will be made available by the authors, without undue reservation.
