# Supplementary material for: Heart Muscle Microphysiological System for Cardiac Liability Prediction of Repurposed COVID-19 Therapeutics
Source: Front Pharmacol. 2021 Aug 4;12:684252. doi: 10.3389/fphar.2021.684252 (PMC8378272; doi:10.3389/fphar.2021.684252)
Supplement: Supplementary file 1 [file DataSheet1.pdf]

Heart Muscle Microphysiological System for Cardiac Liability Prediction of Repurposed COVID19 Therapeutics

Supplementary Material

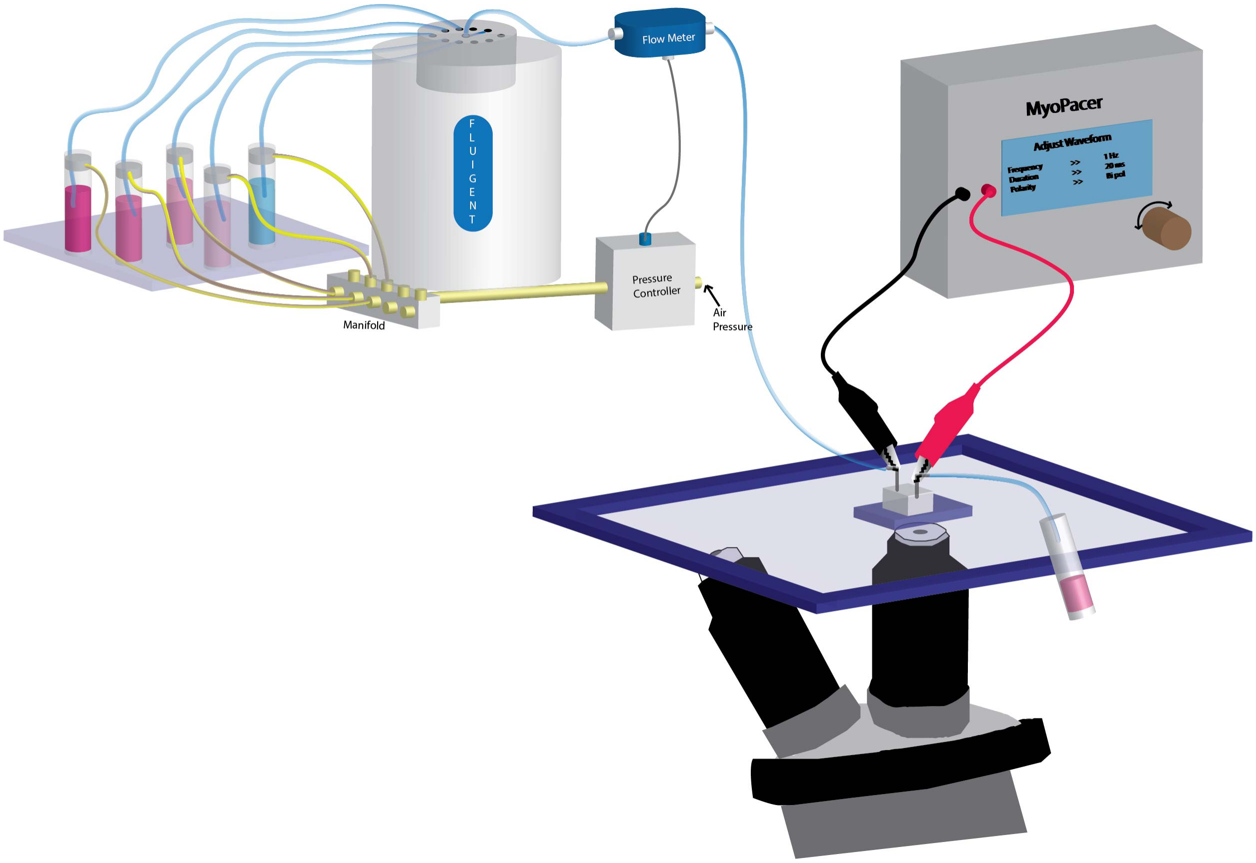

Supplementary Figure 1. Illustration of the experimental setup for automated dose escalation studies. A pressure driven pump system (Fluigent) comprising a FlowEZ pressure control unit (up to 345mbar output pressure), a flow sensor (size M) and a 10-way selection valve (M-switch) was used for controlled and automated perfusion of increasing drug doses (illustrated as vials with pink to blue gradient) through the cardiac MPS. A feedback loop with the flow sensor enabled precise regulation of the volume flow throughout the experiment. The Fluigent system was programmed via the Microfluidics Automation Tool (version 2018). The MPS were imaged on a 37°C heated microscope stage and connected to a field stimulator (ION OPTIX Myopacer) using alligator clips. A 20ms biphasic pacing pulse was sent to the tissue at about 125% of the minimum capture voltage (around 3V) at 1 or 1.5Hz depending on the frequency at which the tissues paced. At the outlet, an eppendorf tube was connected for effluent collection and subsequent proteomics analysis.

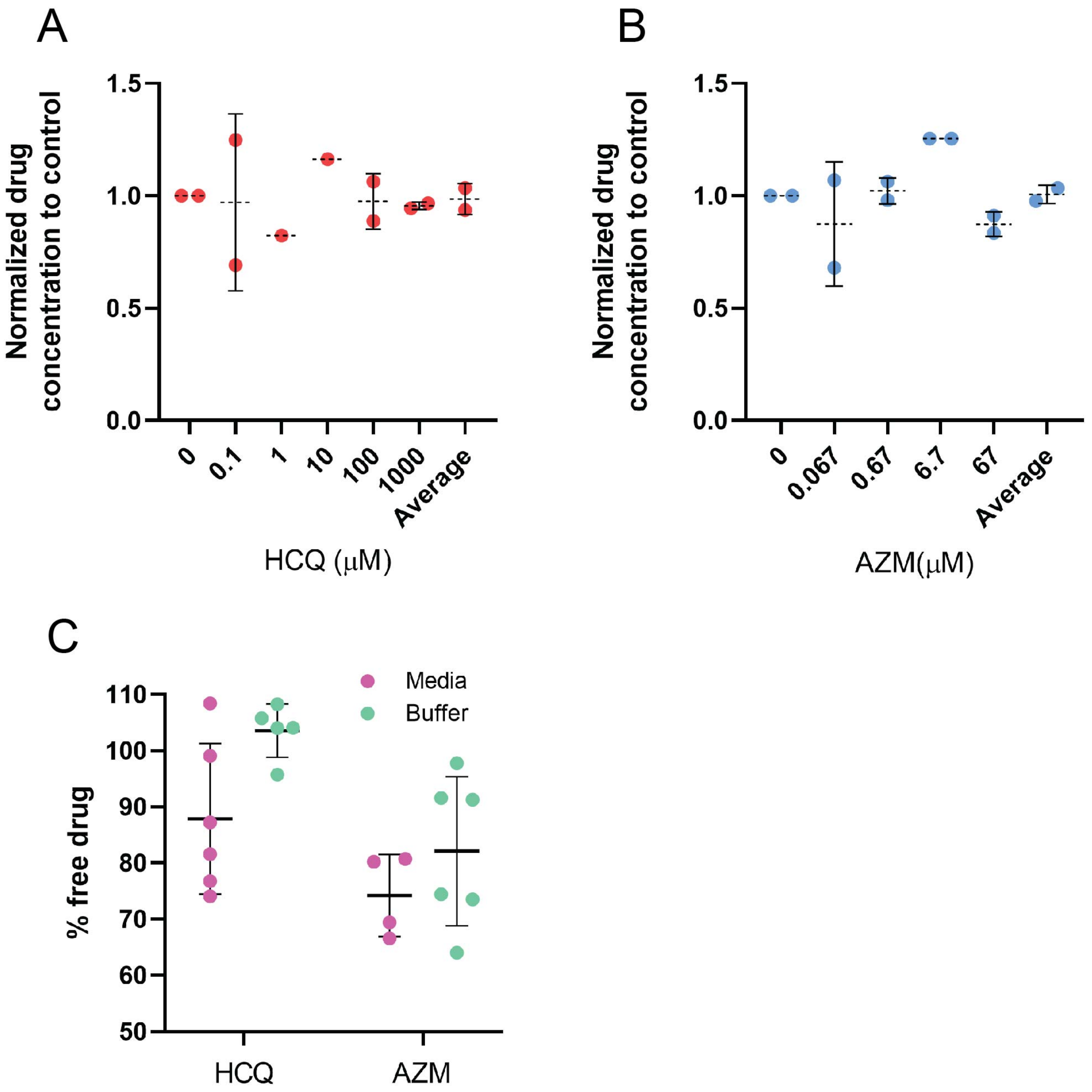

Supplementary Figure 2. Drug absorption in the device components and free drug in media. Absorption studies for dose escalation exposure to HCQ alone (A), or AZM alone (B). A dose escalation study without cells was setup as shown in **Supplementary Fig. 1**. Effluent collection was started 3 minutes after switching to the respective dose to allow washout of the previous dose and stopped as the pump switched to the next higher dose. Graphs show normalized values to HCQ and AZM controls of the respective dose without perfusion through the microfluidic system. No significant drug loss was observed at any dose. (C) Graph showing the percentage of drug (HCQ or AZM) free fraction in either experimental media (maturation media) or buffer (blank, isotonic sodium phosphate). Differences between the media and buffer were not significant.

Supplemental Methods

In-house Python script

Post-experiment processing was performed with an in-house Python library. This library has integrated automated background subtraction and normalization, and eliminates bleaching baseline drift by fitting a polynomial to the baseline via half-quadratic minimization (Mazet et al., 2005). The resulting traces were used for quantitative analysis of the action potential by calculating metrics such as 80% and 30% action potential duration (APD_{80} and APD_{30}), triangulation ((APD_{80}-APD_{30})/ APD_{80}) and beat rate. Similar metrics were calculated for calcium transient (CaD_{80} and CaD_{30}). Poincaré plots were generated by the same library, by plotting CaD_{80} of each (n^{th}) beat, against CaD_{80} of the preceding beat $(n-1)^{th}$, normalized to the CaD_{80} mean. Identical CaD_{80} values in sequence appear as a single point, stable CaD increase or decrease (anti-arrhythmic) will cluster around the center of the graph and large deviations between successive CaD_{80} (pro-arrhythmic), points will deviate from the center giving rise to disorganized polygons (Hondeghem et al., 2001).

Drug absorption into PDMS

In order to quantify the actual drug concentration available to the microtissues, drug absorption into the device (PDMS, Polydimethylsiloxane, or tubing) was measured. Drug absorption experiments were performed and analyzed via liquid chromatography-mass spectrometry (LC-MS/MS) for HCQ (**Supplementary Fig. 2A**) or AZM (**Supplementary Fig. 2B**). The drug doses were prepared the same way as for the cell experiments. The experimental setup of acute studies was replicated in the absence of cells and the effluent collection was started 3 min after switching to the respective dose. Additional samples from freshly prepared drug doses were collected for controls. All samples were immediately frozen at -80°C.

Drug free fraction in media

The binding of test compounds to plasma proteins is an important factor affecting drug efficacy, metabolism and pharmacokinetic properties. In many cases, drug efficacy is determined by the concentration of free drug (unbound), rather than the total concentration in plasma. If the drug is highly bound to plasma proteins, the amount of drug available to reach the target is reduced. We therefore measured the portion of free drug in the media with RED (rapid equilibrium analysis) assays (Thermo Scientific, 90006) to determine the media-bound versus free fraction of drug following manufacturer's instructions (**Supplementary Fig. 2C**). Briefly, experimental media (maturation media, MM) spiked with test compound was added to the left chamber of a commercial plate based RED (rapid equilibrium dialysis) device. Blank, isotonic sodium phosphate buffer was added to the outer chamber of the RED device and the plate was incubated at 37°C. Aliquots of the buffer and MM were taken at pre-determined time points and the concentration of free and bound test compound was determined by LC-MS/MS analysis.

LC-MS/MS analysis

Samples (10 µL) were chromatographed on a ZORBAX SSHD Eclipse Plus C18 column (3.0 x 50 mm, 1.8 µm, catalog no. 959757-302; Agilent, Santa Clara, CA) with a guard column (2.1 x 5 mm, 1.8

μm, catalog no. 821725-901; Agilent) via an Agilent 1290 Infinity II LC system. Column temperature and the LC flow rate were set at 40°C and 0.4 ml/min. Initial chromatographic condition was maintained at 95% mobile phase A (water with 0.1% formic acid, v/v) and 5% mobile phase B (acetonitrile with 0.1% formic acid, v/v) for one min, then increased to 80% B by 3 min, then to 95% B by 4 min, and then returned to initial condition at 5 min until 8 min for sufficient equilibrium. All MS/MS analyses were performed in positive ion mode with an electrospray ion (ESI) source using an Agilent 6470 triple-quadruple mass spectrometer. The capillary voltage was set at 3500 V. The nebulizer gas pressure and gas temperature were set at 35 psi and 350°C, respectively. The MS/MS transitions, collision energy (CE), and fragmentor energy (FE) were set for the detection of AZM (m/z 749.5→591.4, CE=29 V, FE=160 V) and HCQ (m/z 336.2→247.1, CE=21 V, FE=106 V).

References

Hondeghem, L.M., Carlsson, L., and Duker, G. (2001). Instability and triangulation of the action potential predict serious proarrhythmia, but action potential duration prolongation is antiarrhythmic. *Circulation* 103**,** 2004-2013.

Mazet, V., Carteret, C., Brie, D., Idier, J., and Humbert, B. (2005). Background removal from spectra by designing and minimising a non-quadratic cost function. *Chemometrics and Intelligent Laboratory Systems* 76**,** 121-133.
